# Development and Characterization of Low Temperature Wafer-Level Vacuum Packaging Using Cu-Sn Bonding and Nanomultilayer Getter

**DOI:** 10.3390/mi14020448

**Published:** 2023-02-14

**Authors:** Taehyun Kim, Sangwug Han, Jubum Lee, Yeeun Na, Joontaek Jung, Yun Chang Park, Jaesub Oh, Chungmo Yang, Hee Yeoun Kim

**Affiliations:** 1 National Nanofab Center, Center of IoT Sensor Development, Daejeon 34141, Republic of Korea; 2 National Nanofab Center, Center of Analysis and Characterization, Daejeon 34141, Republic of Korea

**Keywords:** wafer-level packaging, Cu-Sn bonding, nanomultilayer getter, microbolometer

## Abstract

Most microsensors are composed of devices and covers. Due to the complicated structure of the cover and various other requirements, it difficult to use wafer-level packaging with such microsensors. In particular, for monolithic microsensors combined with read-out ICs, the available process margins are further reduced due to the thermal and mechanical effects applied to IC wafers during the packaging process. This research proposes a low-temperature, wafer-level vacuum packaging technology based on Cu-Sn bonding and nano-multilayer getter materials for use with microbolometers. In Cu-Sn bonding, the Cu/Cu_3_Sn/Cu microstructure required to ensure reliability can be obtained by optimizing the bonding temperature, pressure, and time. The Zr-Ti-Ru based nanomultilayer getter coating inside the cap wafer with high step height has been improved by self-aligned shadow masking. The device pad, composed of bonded wafer, was opened by wafer grinding, and the thermoelectrical properties were evaluated at the wafer-level. The bonding strength and vacuum level were characterized by a shear test and thermoelectrical test using microbolometer test pixels. The vacuum level of the packaged samples showed very narrow distribution near 50 mTorr. This wafer-level packaging platform could be very useful for sensor development whereby high reliability and excellent mechanical/optical performance are both required. Due to its reliability and the low material cost and bonding temperature, this wafer-based packaging approach is suitable for commercial applications.

## 1. Introduction

Recently, the size and power consumption of sensors have been greatly reduced due to the emergence of smart sensors combined with ICs and edge sensors with AI functions. For this reason, wafer-level packaging technology is attracting attention; it has already been widely applied to image sensors [1]. However, commercial applications remain limited with microsensors that require complex and precise environmental conditions, such as microbolometers and microgyroscopes, which require interaction with the external environment and internal hermeticity [2]. For example, microbolometer is a type of thermal imaging sensor that operates by detecting changes in temperature. It typically consists of a suspended, microfabricated resistor that is positioned over a thermal isolation structure, such as a vacuum gap or a low thermal conductivity material, that separates it from the substrate. The resistor is connected to a readout circuit, which provides a measure of its resistance. When infrared radiation is incident on the microbolometer, it heats up the suspended resistor. This increase in temperature results in an expansion of the resistor, which changes its resistance. The readout circuit measures this change in resistance, and the resulting signal is processed to generate an image. The amount of change in resistance is proportional to the incident radiation, allowing the microbolometer to measure the temperature of the scene being imaged. Therefore, the performance of the microbolometer depends on the infrared transparency and vacuum conditions, which must be satisfied by its packaging. This makes the packaging of the microbolometer complex and challenging.

The need for reduced sensor size, weight, power and cost has led to a shift from multichip packaging to system-on-a-chip (SoC) packaging. Pixelated sensors, which require high interconnect densities, have transitioned from die-level packaging to wafer-level packaging to satisfy the needs. The trend is converging towards the need for IC-compatible wafer-level vacuum packaging (WLVP) solutions that provide hermetic or vacuum enclosures for SoC smart sensing components. Traditional WLVP approaches like anodic, direct or glass frit bonding are not compatible with IC circuitry, so device and process designers are modifying CMOS-compatible interconnect solutions for WLVP of smart sensors. The most thoroughly researched CMOS-compatible WLVP solutions are Cu-Sn solid-liquid interdiffusion (SLID), Au-Sn eutectic and Au-Au and Cu-Cu thermocompression bonding techniques. The development of a successful WLVP process depends on bonding process and getter materials. Most commercially available wafer bonding process and thin-film getter materials were developed for traditional MEMS bonding techniques and may not be suitable for SoC packages. Improved patterning techniques for thin-film getters and getters with lower activation temperatures are needed to enable the WLVP of smart sensors. The development of CMOS-compatible WLVP could reduce the cost and development cycle for smart sensors, allowing them to meet the demands of the Internet of Things [3]. In general, wafer-level packaging consists of a device wafer and a cover wafer. Device wafers require the deposition of various materials such as bonding layers, optical coatings and getters. As such, the production process is complex [3]. In addition, when sensors are combined with ICs, the processing temperature cannot exceed 400 °C, which is a significant limitation [4].

In the case of the bonding layer, plating and deposition processes have been applied using various binary materials, such as Au-Sn, Cu-Sn, etc., and low-temperature processes and low-priced bonding materials are needed for mass production [5,6]. Recently, Cu-Sn, which is relatively cheap, has been widely used, and various thermal treatment methods in which microstructures were obtained have been reported, leading to increases in reliability [7,8,9,10,11,12]. Kumar S. et al. reports Matano plane-based diffusion model Cu-Sn diffusion couples [7]. The diffusion behavior in Cu-Sn couples was investigated by Yuan Y. et al. within a temperature range of 130–200°C over various annealing periods [8]. The growth of the Cu_3_Sn and Cu_6_Sn_5_ phases was assessed, with the Cu_6_Sn_5_ phase being diffusion-controlled from the beginning, while the Cu_3_Sn phase changed to diffusion-controlled growth later. In addition, the interdiffusion coefficients and activation energies for diffusion were also evaluated. Luu T. et al. characterized the formation of intermetallic compounds (IMCs) during Cu-Sn wafer-level bonding process by developing thermal kinetics models for the thickness of Cu_3_Sn and the amount of Sn converted into IMCs [10]. Wu D. et al. presents a high-density, irreversible Cu-Sn bump eutectic bonding technology for wafers in their research. The technology is founded on the principles of low-temperature bonding of Cu-Sn and testable current conduction, leading to the formation of a dense Cu_3_Sn IMC layer [11]. 

In the case of getter materials, Zr, Co, and Ti NEG getters have been widely used [13,14,15]. Jin Y.F. et al. presents a method to maintain vacuum by combining MEMS fabrication with getter material preparation [14]. The process of coating a thick film of getter material consisting of Zr, V and Fe on Si and glass wafers was studied in detail. Properties such as adhesive strength and sorption capacity of the NEG films were examined. Ferrario B et al. introduces the use of metals like Th, Ce, La, Al, Zr, and Ti in various alloys as getter materials to absorb various gases. These metals have different properties and have been used in different ways to getter specific gases. Zr and Ti have been studied extensively in the last few decades and are commonly used in alloy form as getters for specific gases in various applications [15]. However, most of getter materials have been used with high activation temperature over 400 °C. Therefore the development of new materials is required due to the high formation temperature required with these compounds.

Until now, most studies on the simultaneous application of Cu-Sn bonding and a getter material with actual microsensors have involved processing at temperatures of 300 °C or higher [16,17,18,19,20,21]. This is because the high activation temperature of the getter material deposited on the cover wafer degrades the effectiveness of the low-temperature Cu-Sn bonding material deposited on both sides of the device and on the cover wafer.

In this study, Cu-Sn, which can be bonded at low temperature, was used as a bonding material, and a nano-multilayer thin film getter that can be activated at low temperature was applied to realize the wafer-level packaging of a microbolometer. By observing changes in the microstructure at different temperatures, pressures, and times during the bonding process, microstructure optimization of the bonding materials was realized. The mechanical reliability was evaluated under each condition. In addition, we present a method to evaluate the direct degree of vacuum inside the package by measuring the thermoelectric properties of microbolometer pixels, rather than the conventional SAM [22] or mechanical deformation measurement methods [23].

## 2. Experiment

### 2.1. Materials

Ti, Cu, and Sn as the bonding material and Ti, Ru, and Zr as the getter material were fabricated by e-beam and a thermal evaporation system (BAK-641, Evatec, Trübbach Switzerland). Depending on the heat treatment condition, the layer structure of the bonding material for diffusion analysis was Ti/Cu/Sn (with thicknesses of 0.2/3/2 µm) on an 8” (100) Si substrate. As a getter material, a Ti/Ru/Ti/Zr (with thicknesses of 0.01/0.06/0.5/0.5 µm, respectively) multilayer structure was fabricated by the same evaporation system. Ru provides a nucleation site to form more grain boundaries while also lowering the formation temperature by better forming the columnar structure reported in our previous paper [24]. The evaporated materials used were Ti (99.9995% stated purity), Cu (99.997% stated purity), Sn (99.99% stated purity), Au (99.995% stated purity), Zr (99.5% stated purity), and Ru (99.95% stated purity). The deposition rate was 2~10 Å/s, depending on the material used. Depositions were performed at room temperature and at a base pressure of 5 × 10^−8^ Torr. The microstructure and chemical composition were analyzed by FE-SEM (SU8230, Hitachi, Tokyo, Japan) and attached EDS (Quantax 400(125 eV resolution), Bruker, MI, USA). The crystal structures of bonded interfaces were characterized by TEM (JEM-ARM200F, JEOL, Japan) and EELS (Enfina 1000(1.5 eV resolution), Gatan, VA, USA).

### 2.2. Fabrication Process

**Figure 1:**
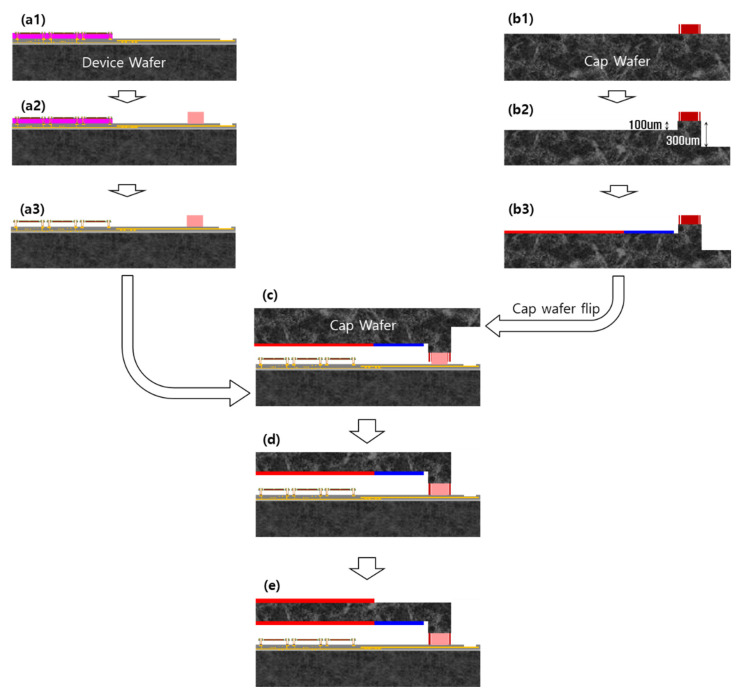
Fabrication process of the device and cap wafer. (**a1**) Device wafer, (**a2**) bonding metal deposition, and (**a3**) membrane release of device wafer. (**b1**) Cap wafer bonding metal deposition, (**b2**) two-step cavity etch by DRIE, and (**b3**) getter, AR deposition by shadow mask. (**c**) Align bonding of device and cap wafer, (**d**) pad opened by back-grinding, and (**e**) AR deposition of the top surface.

The wafer level packaging of the microbolometer consisted of a device wafer and a cap wafer. Bonding metal was deposited on the device wafer, and the bonding metal, antireflective (AR) material, and getter were deposited on the cap wafer (size: 8”). Figure 1 shows the manufacturing process of the device wafer and the cap wafer. 

The fabrication process of the device wafer is as follows. A 360 × 240 microbolometer focal plane array (MBFPA) with 12 µm pixels was integrated using an amorphous silicon material compatible with the CMOS process. Figure 1a1~a3 illustrates the fabrication process flow for a single microbolometer pixel with an air gap. The pixel is designed to maximize LWIR absorption and includes an IR-sensitive layer of boron-doped amorphous silicon (a-Si) isolated thermally from the reflective mirror. The bottom mirror and lower electrode metal layer, made up of Ti/Al/TiN with thicknesses of 10/800/15/2 nm, were deposited using sputtering equipment (Endura 5500, AMAT, CA, US) and patterned by etching on the SiO_x_/Si substrate. A dielectric layer and a sacrificial amorphous carbon layer (ACL) were deposited using a plasma-enhanced chemical vapor deposition (PECVD, P-5000, AMAT, CA, US) system. A sub-passivation layer of SiN_x_ was deposited and patterned to open a via hole through the passivation, the sacrificial layer, and SiO_x_ on the pad metal. A 300-nm-thick TiN layer was plated to fill the via holes and connect the thermistors to the readout integrated circuit (ROIC). A 15-nm-thick TiN absorption layer was deposited with a patterned spacing of 400 nm. A 30-nm-thick SiN_x_ layer was deposited to fabricate a membrane with a co-planar structure, followed by a 100-nm-thick boron-doped a-Si layer serving as the temperature sensing material. An additional passivation layer of SiN_x_ was deposited to complete the sandwich structure, and the serpentine leg and bolometer membrane were defined through a dry etching step. The ACL sacrificial layer was then removed using O_2_ plasma stripping to suspend the microbolometer structure. The thickness of the passivation layers between the bottom and top layers was 110 nm, creating a thinner bolometer membrane with lower heat capacity to minimize thermal conductance. The fabricated schematic structure of the microbolometer is shown in (a1) of Figure 1.

**Figure 2:**
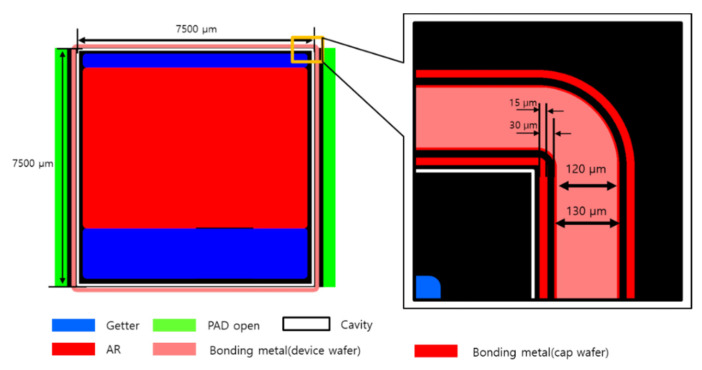
Single die layout of microbolometer cap wafer and enlarged WLP area. Layer colors are the same as in Figure 1.

**Figure 3:**
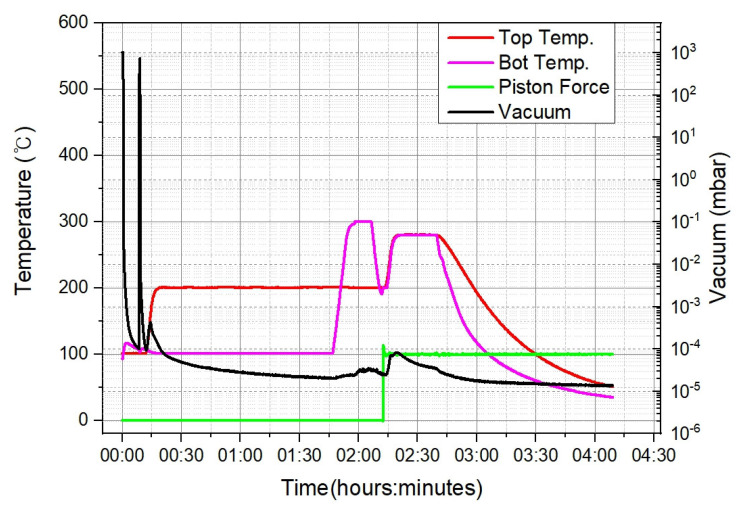
Parameter (temperature, pressure, time) profiles during the bonding process.

The bonding metal of the device wafer was composed of Ti/Cu/Sn (with thicknesses of 0.2/3/3 µm, respectively), which was deposited by lift-off lithography and thermal evaporation. The cap wafer cavity was dry-etched in a two-stage structure, and the electrode was opened by back-grinding after bonding. The bonding metal of the cap wafer was deposited by thermal evaporation with Ti/Cu/Au (thicknesses of 0.2/3/0.02 µm, respectively) without Sn to form the getter. Figure 2 shows a schematic of the WLP process. The width of the bonding surface was 120 µm; a dam of 30 µm is shown on both sides. The getter was deposited by thermal evaporation in the side areas of the cavity of the cap wafer. Since we wanted to deposit this inside the cavity with a vertical slope with a depth of 100 µm (Figure 2), a Si shadow mask with alignment accuracy of several tens of um was applied. After getter activation of the cap wafer at 300 °C for 30 min, bonding was carried out under a vacuum of 10^−5^ mbar (Gemini 200, EVG, Ebensee, Austria). Figure 3 shows the temperature, vacuum, and bonding force over time during the bonding process. A minimum bonding force is required to balance between the surface oxide break and wafer damage. To optimize the bonding strength, the bonding force was changed to 2.5, 5, and 10 kN.

The bonding strength was measured using five specimens for each condition using a shear tester (Dage 4000, Dage Precision Industries, Cambridgeshire, UK). The thermoelectric characteristics of the microbolometer test pixel were evaluated using a probe station (M6VC, MSTECH, Hwasung, Korea), as described in previous reports [25,26].

## 3. Results and Discussion

### 3.1. IMC Growth Behavior of Cu-Sn Binary System

**Figure 4:**
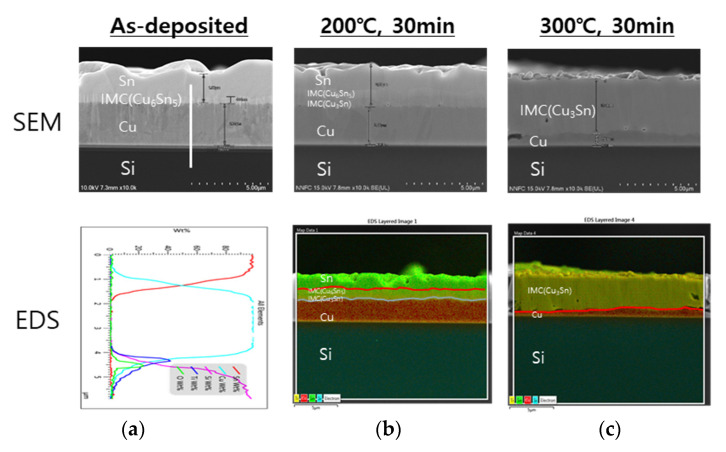
SEM and EDS analysis results of Cu-Sn diffusion couples on a Si substrate (**a**) as-deposited or with annealing (**b**) at 200 °C or (**c**) 300 °C.

**Figure 5:**
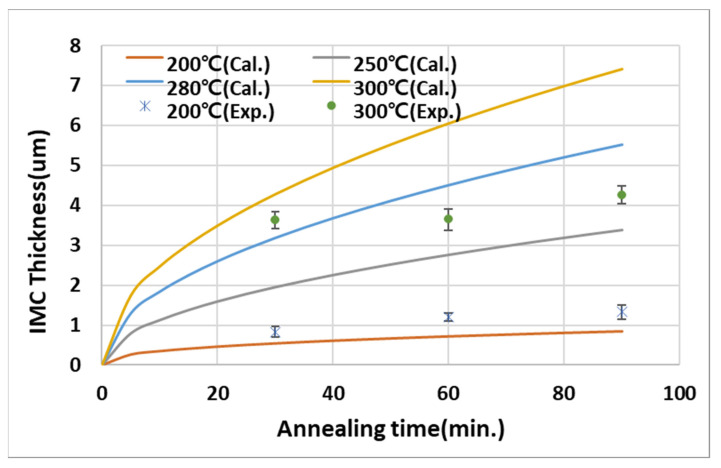
Cu_3_Sn growth behavior with annealing time. Solid lines were calculated using Equation (1); dots are experimental values.

Figure 4 shows the microstructures of the as-deposited and heat-treated at 200 °C or 300 °C samples in the Cu-Sn binary system. The first generated phase at the interface during the deposition process was Cu_6_Sn_5_(η), which transformed into Cu_3_Sn(ε), depending on the heat treatment temperature and time. To ensure reliability, the final microstructure after wafer bonding required a Cu/Cu_3_Sn structure in which Sn was completely consumed, and an appropriate thickness and thickness ratio of Cu and Sn had to be designed. In general, the growth of the intermetallic compound (IMC) varied according to the Arrhenius equation.

(1) $$y_{t}^{2}-y_{0}^{2}=k_{0}\exp\left(\frac{-Q}{RT}\right)t^{2n}$$

where *y_0_* is the initial thickness of IMC, *y_t_* is the thickness at time *t*, *k_0_* is the diffusion coefficient, *R* is the ideal gas constant, *T* is the temperature, *Q* is the activation energy, and *n* is the growth index. Figure 5 shows a comparison between the Arrhenius model and the experimental results of the thickness change (*y_t_*) of the Cu_3_Sn phase over time and with different heat treatment temperatures in the Cu-Sn binary system using the values reported previously [10]. In this experiment, the initial thicknesses of the deposited Cu and Sn were 3um and 2um. Two phases coexisted at 200 °C below the melting point of Sn, and it was difficult to completely transform the Cu_6_Sn_5_ phase to the Cu_3_Sn phase, even if the thermal treatment time increased. However, it was observed that the Cu_3_Sn phase was initially generated at 300 °C, and there was no significant change in thickness, even if the time increased. Therefore, it was possible to obtain a Cu/Cu_3_Sn microstructure when the initial thickness ratio of Cu and Sn was set to exceed 1:1.3 [10], i.e., the minimum stoichiometric thickness ratio of Cu and Sn to obtain the Cu_3_Sn phase, and heat treatment was performed at 250–280 °C.

### 3.2. Microstructure and Mechanical Properties of Bonded Interface

**Figure 6:**
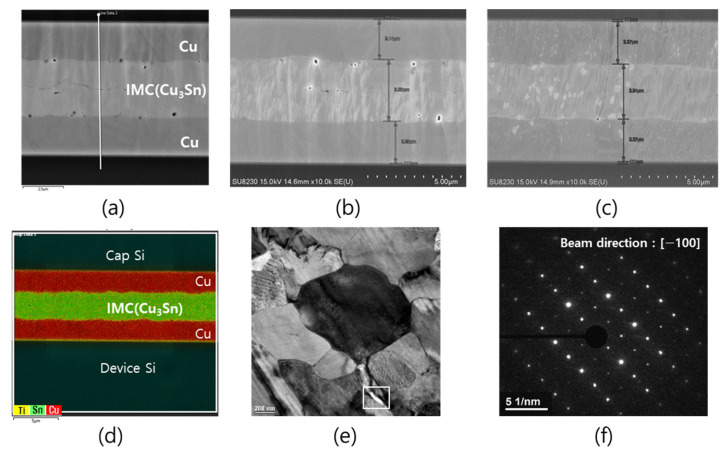
Cross sectional SEM micrograph of the bonded interface at bonding forces of (**a**) 2.5 kN, (**b**) 5 kN, and (**c**) 10 kN. (**d**) EDS map, (**e**) TEM micrograph, and (**f**) diffraction analysis results of sample (**c**) showing that Cu_3_Sn IMC has an orthorhombic crystal structure with lattice parameters of a = 5.49, b = 4.32, c = 4.74 Å [30,31].

**Figure 7:**
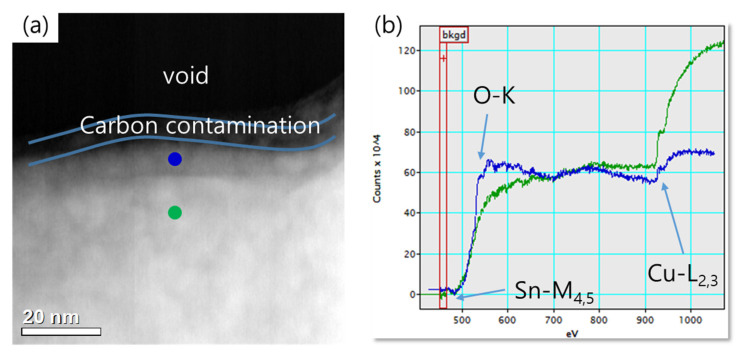
(**a**) TEM micrograph and (**b**) EELS spectrum of void area (square) in Figure 6e; blue for the interface and green for the underlayer. Carbon contamination originated from sample the preparation. The energy level of each element was 532 eV, 485 eV, and 935 eV for O-K, Sn-M4,5, and Cu-L2,3 [32,33].

Figure 6 shows the microstructure of the bonding cross-section according to the pressing force. It shows microstructures consisting of residual Cu and Cu_3_Sn, as analyzed by EDS and TEM. Most of the voids were observed mainly at the Cu/Cu_3_Sn interface, while discontinuous voids were observed at the original bonding interface when low pressure was used. In general, in Cu-Sn TLP bonding, voids may occur inside the original bonding interface, Cu/Cu_3_Sn interface, and Cu_3_Sn interface [27,28,29]. Void areas in the original bonding interface area were analyzed by EELS with high energy resolution (1.5 eV), as shown in Figure 7. This figure shows that Cu-Sn-Ox existed near the void of the original interface. Even though the top surface of the bonding metal was protected by a thin Au layer, it can be seen that the void at the bonding interface was mainly caused by the surface oxidation of Cu and Sn that may occur in deposition and other processes. The size of the void decreased as the pressure increased.

**Figure 8:**
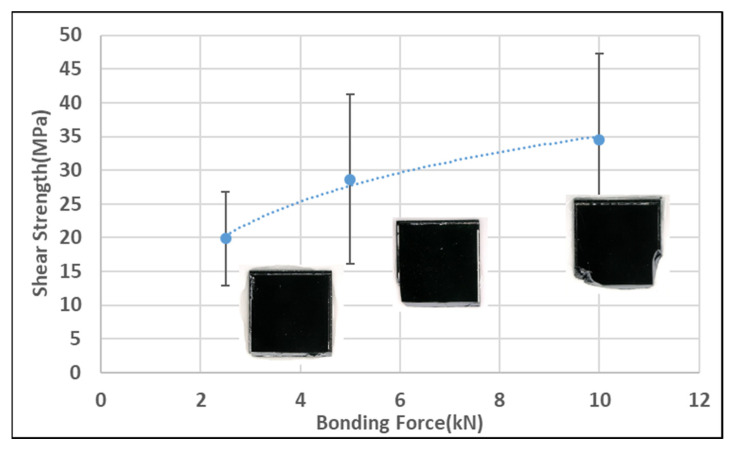
Die shear strength and fractured shape depending on the bonding force.

Figure 8 shows the die shear strength according to the bonding force. As the force increased, the shear strength increased due to the decrease in the size of the void, as shown in Figure 6. The average shear strength was 20 to 35 MPa, which was similar to the previously reported results [17,18]. 

### 3.3. Vacuum Characteristics

**Figure 9:**
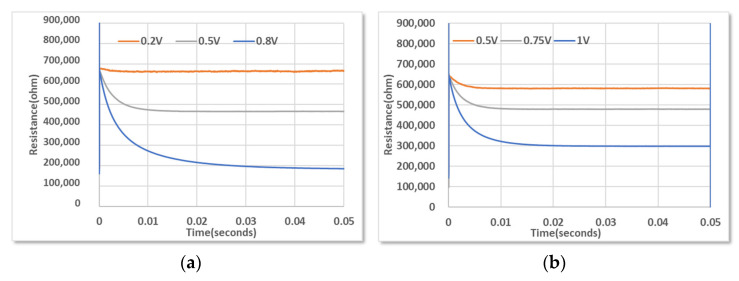
Resistance change with time at (**a**) 20 mTorr and (**b**) 100 mTorr under pulsed bias condition (pulse width: 30 µs).

Figure 9 shows the change of resistance of a microbolometer over time under controlled vacuum conditions. The measurement systems and evaluation methods were presented in detail in previous reports [25,26]. The degree of vacuum affects the heat transfer of the microbolometer. A microbolometer follows the same heat transfer equation as a Pirani gauge, i.e., with heat transfer by conduction and radiation determined by the structure, independent of the vacuum level; only the convection is related to the vacuum level [30,31]. Equation (2) is the heat transfer equation of the microbolometer in the absence of an external infrared source [34].

(2) $$C\frac{d\left(\Delta T\right)}{dt}+G_{e}\Delta T=P_{V}$$

where *C* is heat capacity, *Ge* is the effective thermal conductivity, Δ*T* = *T* − *T_0_* is the temperature increment, and *P_V_* is the power dissipation of the microbolometer. The thermal conductivity under saturation is expressed as follows:

(3) $$G_{e}=\frac{V^{2}R_{s}}{\Delta T}=G_{gas}+G_{solid}+G_{rad}$$

where *V* is the bias voltage, *Rs* is the saturated resistance, *G_gas_* indicates convection, *G_solid_* indicates conduction, and *G_rad_* indicates radiation. When there was no external input (*P_V_* = 0), the temperature change from Equation (2) is expressed as follows:

(4) $$\begin{array}{cc} \frac{d\left(\Delta T\right)}{dt} & =-\frac{G_{e}}{C}\Delta T\\ \Delta T & =\Delta T_{0}exp\left(-\frac{G_{e}}{C}t\right),\;\tau_{e}=\frac{C}{G_{e}} \end{array}$$

where *τ_e_* is the thermal time constant. Under constant voltage, the temperature of the electrical resistor changed exponentially and was eventually saturated. The thermal time constant could be obtained with a heating or cooling curve. The thermal time constant was defined as the time at which 63.2% of the saturated value occurred, i.e., a natural logarithmic characteristic. Therefore, temperature change with time could be determined as follows:

(5) $$\begin{array}{cc} & T=T_{0}+\left(T_{1}-T_{0}\right)exp\left(-\frac{G_{e}}{C}t\right)\;\mathrm{for}\;\mathrm{cooling}\;\\ & T=T_{0}+\left(T_{1}-T_{0}\right)\left(1-exp\left(-\frac{G_{e}}{C}t\right)\right)\;\mathrm{for}\;\mathrm{heating} \end{array}$$

Microbolometers use resistors whereby the temperature correlates to resistance according to temperature coefficient of resistivity (TCR) *α*. We used amorphous Si as a resistor; its TCR has the negative value, which is typical of a semiconductor, as described in Equation (6). The thermoelectrical characteristics of amorphous Si and the experimental results were reported in [35].

(6) $$\alpha =\frac{1}{R}\frac{dR}{dT},\;R\left(T\right)=R_{0}\left(T_{0}\right)exp\left[\alpha T_{0}{}^{2}\left(\frac{1}{T}-\frac{1}{T_{0}}\right)\right]$$

Therefore, the change in resistance over time was the same as the change in temperature, and the vacuum level could be evaluated through the change in the thermal time constant. From the heating curve shown in Figure 9, the thermal time constant (*τ*) was obtained by empirical decay fitting, and the change according to the vacuum level (*P*) was measured. Table 1 shows the thermoelectrical parameters including the thermal time constant and electrical variables measured at each vacuum level. The change in the thermal time constant depending on the vacuum level showed similar behavior as the changes in thermal conductivity predicted using Equation (4).

The above measurement enabled us to derive the heat capacity of the structure of the microbolometer, i.e., the sum of the heat capacity of each layer, consisting of the membrane ($C=\sum A_{i}t_{i}\rho_{i}c_{i}$, where *A* is area, *t* is thickness, *ρ* is density, and *c* is the specific heat), as listed in Table 2. The calculated heat capacity ranged from around 1.6 to 2.8 × 10^−10^ (J/K) and showed similar results to the measured values, proving the validity of this measurement method.

**Figure 10:**
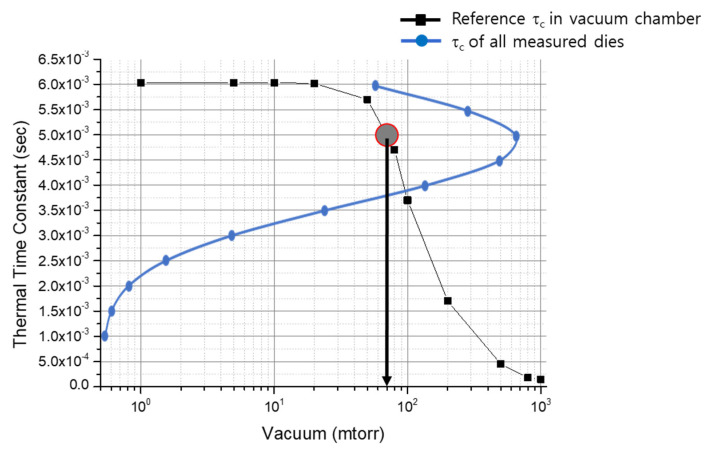
Distribution of thermal time constant of wafer-level packaged samples overlaid on a τ-P curve.

**Figure 11:**
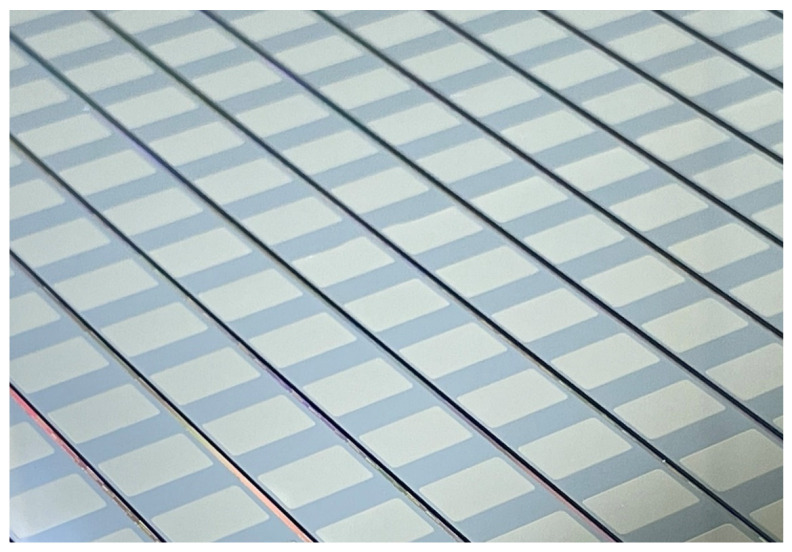
Wafer-level packaged wafer with opened electrical pad.

In Figure 10, the measured thermal time constants of all wafer-level packaged dies are overlaid on the reference curve. The thermal time constants have the centered value of 4.8 ± 1.3 ms, which shows the data distribution around 50 mTorr, thereby satisfying the microbolometer requirement. Data distribution was mainly affected by the uniformity of the fabrication process, which needs further development. Figure 11 shows the final wafer-level packaged wafer (8” in size). This wafer-level packaging approach not only enhances the possibility of commercializing low-temperature and low cost sensors but also provides an effective tool for testing the reliability of packaged samples at the wafer-level.

## 4. Conclusions

Wafer-level packaging for low-cost and low-temperature microbolometers was implemented using Cu-Sn bonding materials and nano-multilayer getters. After bonding, the packaging was composed of Cu/Cu_3_Sn/Cu and showed a microstructure without voids. The possibility of the commercial application of the proposed method was shown, i.e., bonding characteristics similar to those of Au-Sn were achieved, and a processing temperature of 300 °C or lower was found to be suitable. Since the evaluation of the degree of vacuum using microbolometer pixels does not require the use of a separate device, it provides an easy way to evaluate the reliability of wafer-level packaging. The manufactured wafer-level packaging showed a vacuum degree of 50 mTorr or less. As all processes including testing were possible at the wafer-level, sensor development was achieved easily, because the consistency of the root cause and failure relationship was always maintained.

## Figures and Tables

**Table 1:** Calculation of thermoelectrical parameters from the thermo-resistance curve (Figure 10) based on vacuum pressure.

Thermoelectrical Parameter	P = 300 mTorr	P = 100 mTorr	P = 20 mTorr
Initial resistance (*R_0_*, kΩ)	626	576	623
Saturated resistance (*R_t_*, kΩ)	526	394	183
Bias Power (W)	1.02 × 10^−6^	8.5 × 10^−7^	1.02 × 10^−6^
Thermal time constant (ms)	1.35	3.31	6.13
Thermal conductance (*Ge*, W/K)	1.6 × 10^−7^	6.7 × 10^−8^	3.63 × 10^−8^
Heat capacity (*C*, J/K)	2.16 × 10^−10^	2.23 × 10^−10^	2.23 × 10^−10^

**Table 2:** Layers and thermal properties [36] of the microbolometer pixel membrane.

Layer Material	Volume (m^3^)	Density (g/m^3^)	Specific Heat (cal/gK)
a-Si as resistor	3.0 × 10^−17^	2.3	0.16
TiN as electrode	3.3 × 10^−18^	5.2	0.14
SiNx(PECVD) as dielectric	4.8 × 10^−17^	3.1	0.17~0.36 [37]

## Data Availability

Not applicable.
